# The Link between Pediatric Obstructive Sleep Apnea (OSA) and Attention Deficit Hyperactivity Disorder (ADHD)

**DOI:** 10.3390/children8090824

**Published:** 2021-09-19

**Authors:** Gino Luis Urbano, Bea Janine Tablizo, Youmna Moufarrej, Mary Anne Tablizo, Maida Lynn Chen, Manisha Witmans

**Affiliations:** 1 Ateneo School of Medicine and Public Health, Pasig 1604, Philippines; 2 De La Salle Medical and Health Sciences Institute, Dasmariñas 4114, Philippines; beatablizo@gmail.com; 3 Valley Children’s Hospital, Madera, CA 93636, USA; ymoufarrej@valleychildrens.org (Y.M.); mtablizomd@gmail.com (M.A.T.); 4 Stanford Children’s Health, Lucille Packard Children’s Hospital, Palo Alto, CA 94304, USA; 5 Seattle Children’s Hospital, Seattle, WA 98105, USA; maida.chen@seattlechildrens.org; 6 Stollery Children’s Hospital, Edmonton, AB T6G 2B7, Canada

**Keywords:** obstructive sleep apnea, sleep-disordered breathing, ADHD, sleep disorder, attention

## Abstract

Obstructive sleep apnea (OSA) is a form of sleep-disordered breathing that affects up to 9.5% of the pediatric population. Untreated OSA is associated with several complications, including neurobehavioral sequelae, growth and developmental delay, cardiovascular dysfunction, and insulin resistance. Attention-deficit/hyperactivity disorder (ADHD) is among the neurobehavioral sequelae associated with OSA. This review aims to summarize the research on the relationship between OSA and ADHD and investigate the impacts of OSA treatment on ADHD symptoms. A literature search was conducted on electronic databases with the key terms: “attention deficit hyperactivity disorder” or “ADHD”, “obstructive sleep apnea” or “OSA”, “sleep disordered breathing”, and “pediatric” or “children”. Review of relevant studies showed adenotonsillectomy to be effective in the short-term treatment of ADHD symptoms. The success of other treatment options, including continuous positive airway pressure (CPAP), in treating ADHD symptoms in pediatric OSA patients has not been adequately evaluated. Further studies are needed to evaluate the long-term benefits of surgical intervention, patient factors that may influence treatment success, and the potential benefits of other OSA treatment methods for pediatric ADHD patients.

## 1. Introduction

Obstructive sleep apnea (OSA) is a form of sleep-disordered breathing (SDB) caused by a complete or partial obstruction of the upper airway, resulting in altered gas exchange and/or sleep disruption. Major risk factors for the development of OSA in children are adenotonsillar hypertrophy and obesity. Other risk factors that have been identified include prematurity, asthma, low socioeconomic status, and abnormal craniofacial features [1,2]. Manifestations of OSA include nocturnal symptoms such as snoring, pauses in breathing, enuresis, and diaphoresis during sleep and may lead to other serious health-related complications [3]. Studies have shown the prevalence of OSA in pediatric patients to be anywhere from 1.2% to 9.5% with a peak at two to six years of age [4,5].

Phenotypic presentation of OSA exists on a wide spectrum and is influenced by multiple factors such as age, ethnicity, and socioeconomic status. For this reason, diagnosis and treatment thresholds and outcomes vary for each child [6]. The American Academy of Pediatrics (AAP) recommends screening for snoring and other SDB symptoms during all well-child visits [7]. A comprehensive evaluation of all sleep-related symptoms is indicated for children who have reported poor sleep quality. Furthermore, polysomnography (PSG) is recommended in children who screen positive for SDB to determine the presence of OSA. Untreated pediatric OSA has been associated with learning and behavioral problems as well as growth and developmental delay. Other complications of OSA include cardiovascular dysfunction and metabolic sequelae [8]. Attention deficit and hyperactivity disorder (ADHD) specifically has been linked to OSA in children [9]. Research has shown that children with SDB, particularly OSA, display ADHD symptoms more often than those without SDB symptoms [10].

ADHD is characterized by a pattern of inattention, hyperactivity, and impulsivity that interferes with daily functioning in more than one setting [11]. The global prevalence of the condition in children is estimated to be approximately 5% [12]. The disorder may affect language, motor, or social development and is associated with difficulty focusing and learning, disorganization, poor comprehension, and irritability. The pathogenesis of ADHD is not explicitly known but is thought to consist of primary factors (genetics, neuroanatomy, and catecholamine metabolism) as well as secondary environmental factors (heavy metal and chemical exposure). Other suspected risk factors of ADHD include substance and medication use during pregnancy, nutrition, lifestyle and psychosocial factors, and sleep disturbances such as OSA [13,14].

Current literature shows bidirectional associations between OSA and ADHD. This review aims to summarize the most recent and available literature on the relationship between OSA and ADHD and investigate the impacts of OSA treatment on ADHD symptoms.

## 2. Methods

A PubMed, EBSCO, and Google Scholar database search was conducted using the terms: attention deficit hyperactivity disorder or ADHD, obstructive sleep apnea or OSA, sleep disordered breathing, and pediatric or children. References cited in selected articles were reviewed. Literature on OSA and ADHD, individually, was reviewed as well for supplemental purposes. Articles referenced for the purpose of this review were published between March 2002 and August 2021. The authors reviewed eligible articles that met criteria and excluded articles with no relevance, case reports, and non-English language studies.

## 3. Results and Discussion

### 3.1. Relationship between ADHD and OSA

The relationship between ADHD and OSA appears to be reciprocal, each exacerbating the symptoms of the other. ADHD symptoms overlap with the diagnosis of OSA, with attention deficits reported in up to 95% of pediatric OSA patients [9]. Obstructive sleep apnea is hypothesized to be one of the five sleep phenotypes associated with ADHD, the four others being hyperarousal, delayed sleep onset insomnia, restless legs syndrome/periodic limb movements during sleep, and sleep EEG epileptiform discharges [15]. A 2018 study done by Miano et al. sought to further investigate this hypothesis. Thirty drug-naïve, pediatric patients with ADHD underwent a sleep assessment involving sleep questionnaires, actigraphy recording, and nocturnal video PSG followed by morning multiple sleep latency testing (MSLT). Twenty-eight of thirty participants were found to have sleep disorder comorbidities, with OSA being found in 15 participants, and all children slept less than the normal duration for their age [16]. Precenzano et al. (2016) found that children with OSA scored higher than control groups in hyperactivity, ADHD index, restlessness–impulsivity, emotional instability, Diagnostic and Statistical Manual of Mental Disorders (DSM)-IV inattention, DSM-IV hyperactivity and impulsivity, and total DSM-IV ADHD [17]. Conversely, OSA was observed in 20–30% of ADHD patients [9].

Wu et al. (2017) investigated the relationship between allergic rhinitis, ADHD, OSA, and age. ADHD was significantly more common in children aged four to five years old with severe adenoid hypertrophy as compared to those without. Allergic rhinitis was positively associated with OSA and ADHD in this age group. In children aged six to 11 years old, ADHD was observed more in children with severe tonsillar hypertrophy as compared to those without. It is difficult to know whether systemic inflammation is causing the resulting adenotonsillar hypertrophy and thus resulting in ADHD-type symptoms or underlying genetic or environmental factors are exacerbating both ADHD and OSA symptoms. The incidence of ADHD also increased with age, which may be linked to the effects of OSA-induced hypoxia on brain development and function [18]. This suggests that the duration and severity of OSA result in more cognitive impairment.

Several studies have sought to explore the relationship between the two conditions. Khadra et al. (2008) demonstrated a complex relationship between regional oxygen saturation (rSO2), apnea–hypopnea index (AHI), and mean arterial blood pressure (MAP) in children with SDB. rSO2 and AHI were negatively associated with SDB, rSO2 and MAP were positively associated with SDB, and AHI and MAP were positively associated with SDB. rSO2 levels increased with age and were higher in females as compared to males. This suggests that males are affected more by ADHD than females. All three variables were strongly associated with SDB severity. The implications of this study suggest that SDB variably affects individuals, and neurocognitive deficits should be expected to vary as well [19]. The severity of nocturnal respiratory impairment has also been demonstrated to be significantly correlated with cases of executive functioning impairment [20]. However, there may be other mitigating factors that influence severity that have not been evaluated, such as environmental factors and nutrition status.

A 2006 study by Gozal et al. observed elevated levels of high-sensitivity CRP (hsCRP) in children with OSA, indicating that hsCRP testing may be useful for monitoring the development of neurocognitive deficits in children with OSA [21]. Another theory poses that the elevated levels of C-reactive protein (CRP) and interleukin-6 due to the systemic inflammatory response caused by OSA correlate with sleep disruption and hypoxemia [22]. Beebe and Gozal (2002) suggest that the hypoxia and sleep disturbance caused by OSA negatively impact the restorative benefits of sleep and cellular and chemical balance, leading to prefrontal cortical dysfunction, which may manifest as overactivity and impulsivity in children [23].

Studies which have linked ADHD and OSA do share other common mitigating factors that may contribute to both conditions. Sleep deprivation, either as fragmented sleep or as reduced total sleep time has also been linked to worse OSA symptoms as well as more severe ADHD behaviors. In addition, stimulant medications also can worsen sleep quality and in turn worsen ADHD behaviors. The elevation in CRP, other inflammatory cytokines and markers are presumed to result in end-organ dysfunction that manifests as ADHD behaviors, OSA symptoms, or comorbid disease (allergic rhinitis, obesity, and cardiovascular disease).

### 3.2. Effects of Adenotonsillectomy on Cognitive Function, ADHD Symptoms, and Quality of Life

Treatment approaches for pediatric OSA include adenotonsillectomy, positive airway pressure therapy, orthodontic measures such as rapid maxillary expansion, and weight reduction for obese patients [24]. Medical management, including anti-inflammatory therapy (intranasal steroids and montelukast), has been used for OSA prior to surgery or as an adjunct post-surgery in children who have residual symptoms of OSA. However, a recent Cochrane review reported insufficient evidence to recommend the use of these medications as treatment for OSA [25]. In addition, the role of these medications in established OSA with ADHD symptoms has not been adequately studied [26].

Adenotonsillectomy is the most common treatment for pediatric OSA [26]. Surgery is the recommended treatment for childhood OSA in the presence of adenotonsillar hypertrophy, including patients with comorbidities such as obesity [24]. Most of the studies involving adenotonsillectomy were prospective cohort studies or retrospective chart reviews with few randomized control trials.

In 2013, the Childhood Adenotonsillectomy Trial (CHAT) study performed a multicenter, single-blind, randomized, controlled trial to evaluate the differences between early adenotonsillectomy versus watchful waiting on neurocognitive and behavioral outcomes in children five to nine years old with OSA. A total of 464 children with OSA confirmed by PSG were randomized into the two groups. Children’s attention and executive function were evaluated using the Developmental Neuropsychological Assessment (NEPSY) at the onset of the study and after seven months. Average NEPSY scores prior to intervention were similar to that of the general population and increased in both groups by the end of the study. This trial did not show any difference in the two main outcomes between groups. The increase was greater in children who underwent adenotonsillectomy but was not significant. However, both caregivers and teachers reported significantly greater improvement in observed behavior on the Conners’ Rating Scale in children who received surgery. Additionally, symptoms of OSA were measured using the Pediatric Sleep Questionnaire Sleep-Related Breathing Disorder scale (PSQ-SRBD) and Epworth Sleepiness Scale (ESS) and were shown to significantly decrease in patients who underwent adenotonsillectomy as compared to watchful waiting. Similarly, quality of life was shown to significantly improve when measured on the PedsQL and OSA-18 scales in children who underwent surgery [27].

Since the CHAT trial, additional randomized clinical trials have sought to evaluate the effect of early adenotonsillectomy in children under the age of five, the age of the youngest patient included in the initial CHAT trial. The 2020 Preschool Obstructive Sleep Apnea Tonsillectomy and Adenoidectomy (POSTA) study by Waters et al. evaluated the effect of early adenotonsillectomy on cognition in children aged three to five years old. They performed a multicenter, randomized, controlled clinical trial in which 190 children with primary snoring or mild to moderate OSA were randomized into two groups. The Woodcock Johnson III (WJ-III) Test of Cognitive Abilities and the Brief Intellectual Ability (BIA) score were used to evaluate intellectual ability at baseline and after 12 months. They found that there was no significant difference in intellectual ability between those undergoing early adenotonsillectomy versus those who did not receive surgery. However, like the CHAT trial, parents reported improvements in sleep, dietary habits, snoring, difficulty breathing during sleep, and daytime sleepiness in children who underwent surgery [28].

While the CHAT trial showed perceived improvement in behavior by caregivers, it did not show statistically significant improvements in cognitive/behavioral scoring. However, there are numerous, smaller albeit non-randomized other studies that have examined the effect of adenotonsillectomy on children with SDB and ADHD which have shown more significant resultssuch as reduced inattention and hyperactivity.

A study done by Al-Zaabi et al. (2018) aimed to evaluate cognitive and behavioral changes among children aged nine to 14 with OSA after adenotonsillectomy. The results of the study showed a 33% improvement in neurocognitive function and general intellectual ability, as well as a 21% reduction in inattention and hyperactivity scores, thus showing evidence of the effectiveness of adenotonsillectomy in improving ADHD-like symptoms among children [29].

A study performed by Ahmadi et al. (2016) evaluated children with ADHD and observed the clinical symptoms before and after adenotonsillectomy. Results showed a 69% improvement in ADHD symptoms one month after surgery and an 86% improvement in ADHD symptoms three months after the procedure [30]. Another study by Amiri et al. (2015) showed that children with SDB, adenotonsillar hypertrophy, and ADHD experienced a significant decrease in ADHD symptoms after adenotonsillectomy at three- and six-month postoperative intervals, with more significant decreases observed at the six-month postoperative follow-up [31].

Dadgarnia et al. (2012) found a significant improvement in ADHD symptoms, especially hyperactivity, six months post-adenotonsillectomy [32]. A 2008 study by Fidan and Fidan further showed the positive impact of adenotonsillectomy on behavioral problems, inattention, and hyperactivity, demonstrating significant decreases in mean scores of ADHD, oppositional-defiant disorder, and conduct disorder symptoms after adenotonsillectomy [33].

In a study done by Wei et al. (2007), SDB-diagnosed children scheduled for adenotonsillectomy were evaluated before and after surgery using the Conners’ Parent Rating Scale-Revised Short Form (CPRS-RS), behavior categories, hyperactivity, and Conners’ ADHD index. ADHD index, cognitive problems, and oppositional behavior all showed significant improvement following surgery [34]. Dillon et al. (2007) showed adenotonsillectomy patients had higher frequencies of DSM-IV diagnoses as compared to a control group. At a follow-up one year later, behavior disorder diagnosis significantly fell to 23.1% among the surgery group [35].

Huang et al. (2007) compared different treatment options for school-age children diagnosed with ADHD and mild OSA. The subjects were divided into three treatment options: methylphenidate (MPH) therapy, adenotonsillectomy, or no treatment. Clinical interviews, ADHD rating scale, CBCL TOVA, and OSA-18 were completed prior to initiation of therapy and 6 months later. Results showed that the surgical and MPH groups improved more than the non-treatment group. The surgical group displayed higher improvements in PSG, questionnaire sleep variables, daytime symptoms, and TOVA subscales (impulse control, response time, and total ADHD score) as compared to the MPH group. This was found to be significantly different between the two groups as well. Hence, researchers suggest that surgical treatment of children with SDB and ADHD may prevent unnecessary long-term MPH use [36].

Weber et al. (2006) investigated ADHD symptoms in children with obstructive ventilatory disorders (OVDs) for improvement in inattention, impulsivity, and hyperactivity after adenotonsillectomy. As some subjects were unable to complete PSG to diagnose OSA, the authors chose to use the diagnosis of OVD instead. The 30 subjects were divided into three groups according to age (four to seven, eight to 10, and >11 years old). Attention, impulsivity, and hyperactivity were assessed preoperatively and six months post-surgery using 30 questions based on DSM-IV criteria. Children aged 8 to 10 displayed the highest improvement in behavior post-surgery. Results were not deemed to be statistically significant due to small sample size, as noted by the authors. In addition, PSG was not used as the basis for diagnosis, and, rather, diagnosis was based on history and physical examination [37].

Chervin et al. (2006), compared children who underwent adenotonsillectomy for indications apart from sleep disorders, including those who tested negative by PSG. Children scheduled for adenotonsillectomy displayed higher baseline ADHD scores as compared to the control subjects. Post-surgery, 50% of the children scheduled for adenotonsillectomy showed improvement in symptoms, no longer meeting the criteria for ADHD [38]. This study illustrates the potential benefits of adenotonsillectomy in children with ADHD symptoms even in the absence of OSA diagnosis.

Another prospective study done by Avior et al. (2004) assessed attention deficit disorder (ADD) in children with OSA scheduled for adenotonsillectomy. Investigators used the test of variables of attention (TOVA) as opposed to the DSM-IV criteria for ADHD diagnosis. Nineteen children were selected for evaluation. TOVA scores, OSA-18, and CBCL/4–8 questionnaires were taken before surgery and two months postoperatively. Results showed significant improvement in TOVA and CBCL/4–8 scores in all children except one [39]. In this study, the short-term benefits of adenotonsillectomy on ADHD symptoms is demonstrated using assessment tools, TOVA, different from those used in the previously mentioned studies.

While there is strong evidence for the short-term benefits of adenotonsillectomy on ADHD symptoms in pediatric OSA patients, the long-term benefits of the surgery require further evaluation. Studies focused on the long-term benefits of adenotonsillectomy are few, often consisting of small sample sizes. One of these studies is Song et al., which sought to investigate the long-term benefits of adenotonsillectomy on behavioral changes in pediatric patients. Twenty children with PSG-documented OSA completed a KOSA-18, the Korean translation of the OSA-18, and ADHD-RS preoperatively and postoperatively. In contrast to the previously discussed studies, the average interval for follow-up was 54.5 months (range 27–98 months). Upon follow-up, the children displayed significantly decreased KOSA-18 and ADHD-RS scores from baseline. While some patients were lost to follow-up, and the small sample size of the study affects its statistical significance, this study provides evidence of the potential long-term benefits of adenotonsillectomy on ADHD symptoms [40]. It is not clear however how many continue to have ADHD nor if the improvement is permanent since OSA has been known to recur in some children post-adenotonsillectomy. A prospective study by Mitchell et al. evaluated the long-term behavioral changes in children with OSA post-adenotonsillectomy. Twenty-three children with PSG-documented OSA underwent adenotonsillectomy and were assessed using the Behavior Assessment System for Children (BASC). BASC scores were taken preoperatively and again at six, nine, and 18 months postoperatively. The investigators found that the children showed improvement in aggression, atypicality, depression, hyperactivity, and somatization at all follow-up intervals. They additionally found that breathing and oxyhemoglobin desaturation improved in post-adenotonsillectomy patients and suggest that this may have led to the decrease in neurobehavioral [41].

Collectively, these multiple studies support the role of adenotonsillectomy for OSA as improving ADHD symptoms and cognitive problems [42]. The studies above focused primarily on the benefits of adenotonsillectomy on ADHD symptoms which are summarized in Table 1. One meta-analysis was included (2014) capturing some of the research. Despite the trend and associations of ADHD and OSA, and reported improvements after surgery, there is heterogeneity among different studies that were included. Heterogeneity was related to different age groups studied, and the variable baseline characteristics of the participants, absence or presence of a control group, and the thresholds for inclusion or exclusion in the meta-analysis. These variables greatly influenced the effect size of the reported improvement, from very mild to moderate under stringent definitions [42].

Research on the benefits of other OSA therapeutic interventions on ADHD symptoms has not been adequately established. One study using continuous positive airway pressure (CPAP) demonstrated significant improvement in attention, working memory, and depressive symptoms in OSA patients [43]. Escobar et al. (2021) hypothesize the use of oral appliance therapy (OAT) for OSA treatment may reduce the symptoms of ADHD, given the relationship between OSA and obstruction of nasal airflow [44]. Further studies are needed to evaluate the effectiveness of the mentioned interventions in treating ADHD in OSA patients.

### 3.3. Linkages between ADHD and OSA across the Lifespan

The studies have shown the association of ADHD and OSA in children and adolescents. There are reported improvements post-surgery. It is not clear whether there is complete resolution of either disorder into adulthood. One study evaluated the relationship between ADHD and OSA in adults. They evaluated 194 adults, including 62% males, and found OSA in 83% of patients, with 60% having moderate to severe OSA. The screen for ADHD was positive in 19% of patients, and there was no association between the presence of ADHD symptoms and the severity of OSA. Despite the selection bias, this study suggests that there continues to be an association between OSA and ADHD even in adulthood. The study did not evaluate the onset of symptoms of either disorder nor reported how many patients had adenotonsillectomy in childhood [45].

## 4. Conclusions

The studies referenced show how adenotonsillectomy may significantly improve the quality of life, learning, and behavioral problems of children with OSA. All levels of SDB from primary snoring to OSA have shown significant short-term improvement in quality of sleep and behavioral disturbance after adenotonsillectomy. Studies evaluating the long-term benefits of adenotonsillectomy have demonstrated its benefits up to 98 months after treatment, but these studies are few and often with small sample sizes. Despite the fact that the large randomized controlled trials showed no neurocognitive differences in children with OSA post-adenotonsillectomy versus watchful waiting in different age groups, clinical practice still favours surgery in children based on favorable parent reports and outcomes. Due to the clinically significant relationship between adenotonsillar hypertrophy, OSA, and ADHD, adenotonsillectomy should be considered as a treatment option for ADHD in pediatric OSA patients. However, it is not yet possible at this stage to predict which populations will benefit from surgery and which will not. Further research must first be conducted to determine if there are any predictors that will help identify those who might benefit from surgery. More research is needed to determine if other OSA treatments such as CPAP use and orthodontics can improve ADHD symptoms. In addition, future research should include larger sample sizes and l For this reason, future research can include larger sample sizes and longer intervals to better understand the long-term benefits of adenotonsillectomy. Finally, research also needs to focus on the childhood origins of adult ADHD and adult OSA.

## Figures and Tables

**Table 1:** Changes in ADHD Symptoms Post-Adenotonsillectomy in Studies of Obstructive Sleep Apnea.

Study, Year	N	Age (Year)	Design Method	OSA/ADHD Assessment	Intervention	Follow-up Interval	Change in ADHD Symptoms and Other Functions Post-AT
Song et al. (2020) [40]	20	3–13	Prospective Cohort Study	PSG, KOSA-18, ADHD-RS	AT	54.5 months (range = 27–98 months)	Significant decreases in KOSA-18 (74.3 to 40.7) and ADHD-RS (17.6 to 10.5) scores
Al-Zaabi et al. (2018) [29]	37	9 to 14	Observational Study	AHI, ODI score, NOD, number of OA incidents, number of CA incidents	AT	3 months	56% reduction in mean AHI score; 21% reduction in mean attention and hyperactivity scores
Ahmadi et al. (2016) [30]	59	6 to 12	Prospective Cohort Study	CRS	AT	1 month and 3 months	Conners’ score decreased significantly one and three months after surgery. The scores at three months were lower than those at one month as well.
Amiri et al. (2015) [31]	53	3 to 12	Prospective Cohort Study	CPRS-R	AT	3 months and 6 months	Significant reduction in ADHD symptoms after surgery three- and six months postoperatively with greater reductions noted at the six-month follow-up
Sedky et al. (2014) [42]	529	0 to 18	Meta-analysis of studies assessing pre- and post-surgery ADHD symptoms	CPRS-R, CTRS, BRIEF	AT	2 to 13 months	Improvement in ADHD symptoms (Hedges’ g = 4.3)
Marcus et al. (2013) [27]	464	5 to 9	Randomized Control Trial	PSG, AHI, OSA-18, PSQ-SRBD, ESS NEPSY, CRS, PedsQL TM	AT or watchful waiting	7 months	Increased NEPSY score, but not significant; Decreased PSQ-SRBD and ESS scores; improvement in Conner’s Rating Scale score improved, PedsQL and OSA-18 scores; OSA resolution in 79% patients; improved AHI in children with baseline severe OSA
Dadgarnia et al. (2012) [32]	35	5 to 12	Quasi-experimental Study	ADHD-RS	AT	6 months	Improvement in ADHD inattention score, hyperactivity, and ADHD combined score
Fidan et al. (2008) [32]	30	4 to 14	Prospective Study	T-DSM-IV-Scale	AT	3 months	Positive impact on behavioral problems (inattention, hyperactivity, oppositional-defiance disorder, and conduct disorder) symptoms
Wei et al. (2007) [34]	117	2 to 17	Prospective Nonrandomized study	PSQ, CPRS-R	AT	6 months	Improvement in sleep and behavior scores
Dillon et al. (2007) [35]	79	5–12.9 years old	Prospective Cohort Study	Frequency of *DSM-IV* attention and disruptive behavior disorder	AT	1 year	ADHD diagnosis in group decreased from 27.8% to 23.1%; 50% of subjects with baseline ADHD no longer met diagnostic criteria
Chervin et al. (2006) [38]	105	5 to 12.9	Case–Control Study	PSG, Neurobehavioral assessment scales (behavior, cognition, psychiatric diagnosis, sleepiness)	AT	1 year	50% noted to have improved symptoms post-AT and no longer met the criteria for ADHD
Weber et al. (2006) [37]	30	4 to 13	Prospective Study	Diagnosis of OVD, Attention/concentration deficit disorders related questions, hyperactivity and impulsiveness adapted from DSM-IV protocol	AT	6 months	Children 8–10 years old experienced the highest improvement post-surgery, but not statistically significant
Mitchell et al. (2006) [41]	23	2.5 to 14.8	Prospective Cohort Study	PSG, BASC	AT	6 months and 9–18 months	Improvement in aggression, atypicality, depression, hyperactivity, and somatization within six months and nine to 18 months post-surgery
Avior et al. (2004) [39]	19	5 to 14	Prospective Study	OSA-18, TOVA, CBCL/4–8	AT	2 months	Improvement in the TOVA scores was significant; Reduction in OSA-18 scores

ADHD: attention deficit and hyperactivity disorder; ADHD-RS: attention deficit and hyperactivity disorder rating scale; AHI: apnea–hypopnea index; AT: adenotonsillectomy; ADHD-RS: ADHD Rating Scale; BASC: Behavior Assessment System for Children; BRIEF: Behavior Rating Inventory of Executive Function; CA: Central Apnea CBCL: Child Behavior Checklist; CRS: Conner’s Rating Scale; CPRS-R: Conners’ Parent Rating Scale- Revised; CTRS: Conners’ Teacher Rating Scale Epworth Sleepiness Scale (ESS); KOSA-18: Korean OSA-18; MPH: Methylphenidate; NEPSY: A Developmental Neuropsychological Assessment; OSA-18: Obstructive Sleep Apnea-18; T-DSM-IV Scale: Turgay DSM-IV-Based Child and Adolescent Disruptive behavioral Disorders Screening and Rating Scale; TOVA: Test of Variables Attention; CBCL/4–8: Achenbach Child Behavior Check List/4–8 attention problems syndrome scales; NEPSY: Developmental Neuropsychological Assessment; NOD: number of desaturations; OA: obstructive apnea; ODI score: oxygen desaturation index score; PSG: polysomnography; OVD: obstructive ventilatory disorders; PedsQL TM: Pediatric Quality of Life Inventory; PSQ: Pediatric Sleep Questionnaire; PSQ-SRBD: Pediatric Sleep Questionnaire Sleep-Related Breathing Disorder scale.

## Data Availability

Not applicable.
